# Burden of hypertensive heart disease attributed to metabolic factors from 1990 to 2021 at global, regional, and national levels: an analysis of the global burden of disease study 2021

**DOI:** 10.3389/fcvm.2025.1572392

**Published:** 2025-05-29

**Authors:** Bo Peng, Yuluo Tu, Cheng Zhou, Gui Xie, Suifa Hu, Jing Zhuang, Kai Huang, Jia Xiong

**Affiliations:** Department of Neurology, Nanchang Hongdu Hospital of Traditional Chinese Medicine, Jiangxi, China

**Keywords:** hypertensive heart disease, high systolic blood pressure, high body-mass index, global burden of disease study 2021, deaths, disability-adjusted life-years

## Abstract

**Background:**

Metabolic factors have an increasing impact on hypertensive heart disease (HHD). This study analyzes the global burden trends of HHD from 1990–2021, focusing on the contributions of high systolic blood pressure (HSBP) and high body mass index (BMI).

**Methods:**

This study, based on the 2021 GBD database, analyzes the trends in deaths, disability-adjusted life-years (DALYs), age-standardized mortality rate (ASMR), and age-standardized DALY rate (ASDR) attributable to HSBP and high BMI from 1990–2021 at global, regional, and national levels. The estimated annual percentage change (EAPC) is used to assess the temporal dynamics of the disease burden, and the relationship between the disease burden and the sociodemographic index (SDI) is explored.

**Results:**

In 2021, deaths and DALYs due to HSBP and high BMI significantly increased compared to 1990. However, the ASMR for HSBP-related HHD [EAPC: −0.68; 95% confidence interval (CI): −0.77 to −0.58] and ASDR (EAPC: −0.90; 95% CI: −0.99 to −0.80) showed a decreasing trend, while the ASMR for BMI-related HHD (EAPC: 0.33; 95% CI: 0.27–0.39) and ASDR (EAPC: 0.15; 95% CI: 0.10–0.21) exhibited an increasing trend. From 1990–2021, the regions with the largest increases in ASMR and ASDR for HSBP-related HHD were Eastern Europe and High-income North America, while the largest increases for BMI-related HHD were seen in High-income North America. Moreover, most of the top 10 countries with the largest increases in ASMR and ASDR due to HSBP and BMI were from Eastern Europe. Additionally, in 2021, China had the highest number of deaths and DALYs globally due to HSBP and high BMI-related HHD. At the SDI level, Low SDI regions had the highest ASMR and ASDR for both HSBP and BMI-related HHD in 2021, with a negative correlation to overall SDI. Furthermore, deaths, DALYs, ASMR, and ASDR due to HSBP and BMI in females were generally higher than in males after the ages of 64 and 54, respectively, with the disease burden mainly concentrated in middle-aged and elderly populations.

**Conclusions:**

Metabolic factors are major risk contributors to HHD, with a disproportionately higher burden of mortality and DALYs observed among older adults, particularly women in later life stages. Given these trends, early identification and intervention in key populations should be prioritized through targeted public health strategies and multilayered interventions to mitigate the global burden of HHD and alleviate its growing strain on healthcare systems.

## Introduction

Hypertensive Heart Disease (HHD) refers to a group of structural and functional cardiac abnormalities caused by sustained elevated blood pressure (1–3). Its clinical manifestations range from asymptomatic or mild chest discomfort and palpitations to severe heart failure and sudden cardiac death, making it one of the leading causes of cardiovascular-related mortality and disability worldwide (4, 5). In recent years, the incidence and mortality of HHD have continued to rise, driven by accelerating population aging, rapid urbanization, and widespread adoption of unhealthy lifestyles. This trend poses a major challenge to public health systems and healthcare resource allocation, particularly in low- and middle-income countries (6–8). Recognizing this growing burden, the World Health Organization has designated HHD as a global priority for cardiovascular disease control, highlighting the urgent need for targeted epidemiological research on its key risk factors.

Among the various contributing factors, metabolic risks—especially high systolic blood pressure (HSBP) and high body-mass index (BMI)—are considered principal drivers of the onset and progression of HHD (9–11). HSBP increases left ventricular afterload, promotes myocardial remodeling, and leads to fibrosis (12), while elevated BMI contributes to metabolic cardiac damage and functional decline through chronic low-grade inflammation, insulin resistance, and dysregulated lipid metabolism (13–15). However, most existing studies have focused on isolated risk factors or limited geographical regions. To date, there remains a lack of comprehensive and multidimensional assessments of HHD burden attributable to HSBP and high BMI, particularly stratified by sex, age group, and sociodemographic index (SDI) at global, regional, and national levels.

To address this gap, the present study utilizes data from the Global Burden of Disease Study (GBD) 2021 (16) to systematically examine the trends in HHD burden attributable to HSBP and high BMI from 1990–2021 across global, regional, and national contexts. By evaluating key metrics including the number of deaths, disability-adjusted life years (DALYs), age-standardized mortality rate (ASMR), and age-standardized DALYs rate (ASDR), and further stratifying results by SDI, this study aims to elucidate the influence of metabolic factors on HHD burden across diverse demographic and socioeconomic profiles. The findings seek to fill a critical gap in the literature and provide an evidence base to inform targeted interventions and data-driven public health strategies.

## Methods

### Data source

The data for this study comes from the GBD 2021 database. The GBD, led by a global health research collaboration network, is a continuous epidemiological study that covers data from 204 countries and regions between 1990 and 2021, evaluating 371 diseases and injuries and 88 risk factors (9, 16). The primary goal is to quantify the burden of diseases, injuries, and risk factors at the global, regional, and national levels, providing standardized scientific data and theoretical foundations for public health policy-making. The GBD database collects data from a variety of sources, including national health statistics, death registration systems, hospital records, disease surveillance, health surveys, and peer-reviewed literature. GBD uses standardized processes, Bayesian hierarchical models to clean and adjust data from multiple sources, overcoming data heterogeneity and potential biases. Furthermore, GBD estimates mortality rates and disease burden through the Cause of Death Ensemble Model, and adjusts for different diseases and risk factors, generating high-precision estimates. In risk factor attribution analysis, GBD uses the Population Attributable Fraction method to assess the contribution of specific risk factors to disease burden.

This study extracts disease burden data related to HHD caused by HSBP and high BMI from 1990–2021, covering 21 global regions, 5 SDI regions, and 204 countries. The primary analysis indicators include deaths, DALYs, ASMR, and ASDR. The study aims to systematically assess the differences in HHD burden across different socio-economic development levels, gender, and age groups, revealing the multidimensional impact of metabolic risk factors on HHD burden and providing scientific evidence for targeted public health policies and interventions. All data were retrieved and extracted through the Global Health Data Exchange (GHDx) platform (https://ghdx.healthdata.org/gbd-2021). Data extraction strictly followed GBD's standardized definitions and analytical processes to ensure consistency in time span, geographic coverage, and data quality. This study is based on publicly available anonymized data and adheres to the Guidelines for Accurate and Transparent Health Estimates Reporting, therefore ethical approval is not required. The standardized research methods and scientific rigor ensure reliable assessment of the HHD burden (17).

### Definitions

In the GBD 2021 classification system, HHD is categorized as a Level 3 disease, with its parent categories being cardiovascular diseases (Level 2) and non-communicable diseases (Level 1) (18). HHD, as a severe cardiovascular disease, holds a prominent position in the cardiovascular disease classification due to its unique pathological mechanisms and clinical manifestations (1). The primary pathological features include left ventricular hypertrophy, myocardial fibrosis, and heart failure, which, in severe cases, can lead to sudden cardiac death. In the International Classification of Diseases (ICD) system, HHD is assigned specific codes, with ICD-9 codes ranging from 402.0–402.91 and ICD-10 codes from I11.0 to I11.9 (19).

In GBD 2021, the two main metabolic risk factors for HHD are explicitly identified as HSBP and high BMI. To evaluate the specific contributions of these risk factors to the HHD burden, GBD uses the theoretical minimum risk exposure level (TMREL) as a framework for risk attribution analysis. According to GBD definitions, the TMREL for HSBP is a systolic blood pressure of 110–115 mmHg, while the TMREL for high BMI is between 20 and 23 kg/m^2^ (9). These thresholds provide a scientific basis for assessing the impact of risk factors on disease burden and lay the foundation for quantifying the attributable portion of the HHD burden.

Deaths are a core indicator of disease severity, representing the number of deaths directly caused by a specific disease or risk factor. It reflects the direct threat of the disease to population health and is a fundamental basis for evaluating the effectiveness of public health policies and the prioritization of resource allocation. DALYs is a composite indicator that combines the years of life lost due to premature death (YLL) and the years lived with disability (YLD) due to the disease. It accounts for both mortality and non-fatal health losses, providing a comprehensive measurement of the overall health burden on individuals and society. DALYs serve as a scientific basis for public health policy and resource allocation.

ASMR is the age-standardized mortality rate, adjusted for the age structure differences across populations, making the mortality rates comparable across different regions or countries. ASMR is usually expressed per 100,000 population and is a commonly used metric for comparing disease burden across regions or over time. ASDR is the age-standardized DALY rate, also typically expressed per 100,000 population. By adjusting for age structure differences, ASDR more objectively and accurately reflects the overall health impact of diseases or risk factors on populations, serving as a key indicator for comparing disease burden across different regions, countries, or time points (20).

SDI is an index that comprehensively measures socio-economic development, consisting of per capita income, average years of schooling for individuals aged 15 and above, and total fertility rate for women aged 15–49. The SDI ranges from 0–1, with higher values indicating higher levels of socio-economic development. Based on SDI scores, GBD classifies global regions into five levels: low, low-middle, middle, middle-high, and high, to analyze the impact of socio-economic development on disease burden (16).

### Statistical analysis

This study is based on the GBD 2021 database and uses key indicators such as deaths, DALYs, ASMR, and ASDR to assess the disease burden of HHD caused by HSBP and high BMI from 1990–2021. To eliminate potential biases from population age structure differences, disease burden data were generated using 95% uncertainty intervals (UI) to ensure accuracy and reliability. We estimated the trend of changes in ASMR and ASDR caused by HSBP and high BMI from 1990–2021 using linear regression models and calculated the EAPC and its 95% confidence intervals (CI). The regression model expression is:

In(ASR)=α+βX+e

Where In (ASR) is the natural logarithm of age-standardized rates, *α* is the constant term, X is the year, β is the regression coefficient representing the trend over time, and e is the error term. Based on the EAPC and its 95% CI, if the EAPC and its lower limit are positive, it indicates an upward trend; if the EAPC and its upper limit are negative, it indicates a downward trend; if both are not significantly different from zero, the trend is considered stable (21). Spearman rank correlation tests were also conducted to explore the relationship between the HHD disease burden and the SDI. All data analyses and visualizations were conducted using R software (version 4.2.2) and the JD_GBDR platform (V2.37) developed by Jingding Medical, with statistical significance set at a *p*-value of <0.05.

## Results

### Global deaths and DALYs

At the global level, the number of deaths from HHD caused by HSBP increased from 713,631.34 in 1990 (95% UI: 576,619.22–794,964.31) to 1,331,780.05 in 2021 (95% UI: 1,120,983.96–1,469,554.44), an increase of 86.62%. Among these, male deaths increased from 298,189.70 (95% UI: 233,306.17–337,161.79) to 557,616.18 (95% UI: 434,698.07–643,407.42), an increase of 87.00%, while female deaths increased from 415,441.64 (95% UI: 318,290.64–478,418.06) to 774,163.86 (95% UI: 618,791.55–878,334.00), an increase of 86.35%. Additionally, DALYs caused by HSBP increased from 15,467,045.95 (95% UI: 12,328,710.33–17,298,409.79) in 1990–25,456,396.75 (95% UI: 21,518,930.89–28,032,522.00) in 2021, an increase of 64.58%. For males, DALYs increased from 7,006,919.92 (95% UI: 5,476,020.35–7,980,659.84) to 11,638,017.78 (95% UI: 9,291,017.73–13,387,788.65), an increase of 66.09%, while for females, DALYs increased from 8,460,126.02 (95% UI: 6,326,289.37–9,914,538.77) to 13,818,378.97 (95% UI: 10,901,617.54–15,512,983.80), an increase of 63.34%. By 2021, the ASMR and ASDR for HSBP-related HHD were 16.31 per 100,000 (95% UI: 13.77–18.01 per 100,000) and 301.51 per 100,000 (95% UI: 255.39–331.84 per 100,000), respectively. From 1990–2021, both showed a downward trend, with EAPC of −0.68 (95% CI: −0.77, −0.58) for ASMR and −0.90 (95% CI: −0.99, −0.80) for ASDR. Notably, in both 1990 and 2021, female deaths and DALYs due to HSBP-related HHD were higher than those for males, but the gender differences in ASMR and ASDR were relatively small.

Deaths from HHD caused by high BMI increased from 240,095.95 (95% UI: 168,850.61–313,373.98) in 1990–594,899.05 (95% UI: 362,924.21–804,913.65) in 2021, an increase of 147.78%. Among these, male deaths increased from 93,612.78 (95% UI: 66,086.33–120,397.78) to 243,060.39 (95% UI: 159,714.60–323,101.44), an increase of 159.64%, while female deaths increased from 146,483.17 (95% UI: 97,562.11–196,209.61) to 351,838.67 (95% UI: 201,115.85–494,197.17), an increase of 140.19%. Furthermore, DALYs from high BMI-related HHD increased from 5,666,016.79 (95% UI: 4,251,059.02–7,069,737.58) in 1990–12,551,752.03 (95% UI: 9,489,123.61–15,451,016.13) in 2021, an increase of 121.53%. Male DALYs increased from 2,414,649.44 (95% UI: 1,794,252.02–3,011,391.35) to 5,646,139.70 (95% UI: 4,247,052.19–6,976,634.91), an increase of 133.83%, while female DALYs increased from 3,251,367.35 (95% UI: 2,294,580.51–4,161,209.41) to 6,905,612.33 (95% UI: 5,009,097.33–8,756,180.55), an increase of 112.39%. By 2021, the ASMR and ASDR for high BMI-related HHD were 7.21 per 100,000 (95% UI: 4.23–9.94 per 100,000) and 147.33 per 100,000 (95% UI: 109.06–183.45 per 100,000), respectively. From 1990–2021, both showed an upward trend, with EAPC of 0.33 (95% CI: 0.27, 0.39) for ASMR and 0.15 (95% CI: 0.10, 0.21) for ASDR. Although the ASMR and ASDR for males were lower than those for females, the growth rate was significantly higher for males than for females (Table 1).

**Table 1:** Global burden of metabolic-related hypertensive heart disease: mortality, DALYs, age-standardized rates, and EAPC, 1990–2021.

High systolic blood pressure					
Measure	1990		2021		EAPC (95% CI)
	Number (95% UI)	ASR, per 100,000 (95% UI)	Number (95% UI)	ASR, per 100,000 (95% UI)	
Both					
DALYs	15467045.95 (12328710.33,17298409.79)	406.34 (328.09,451.22)	25456396.75 (21518930.89,28032522.00)	301.51 (255.39,331.84)	−0.90 (−0.99, −0.80)
Deaths	713631.34 (576619.22,794964.31)	20.91 (17.16,23.22)	1331780.05 (1120983.96,1469554.44)	16.31 (13.77,18.01)	−0.68 (−0.77, −0.58)
Male					
DALYs	7006919.92 (5476020.35,7980659.84)	402.76 (316.83,454.77)	11638017.78 (9291017.73,13387788.65)	301.39 (240.98,345.17)	−0.86 (−0.97, −0.75)
Deaths	298189.70 (233306.17,337161.79)	20.25 (16.02,22.58)	557616.18 (434698.07,643407.42)	15.85 (12.36,18.25)	−0.68 (−0.80, −0.57)
Female					
DALYs	8460126.02 (6326289.37,9914538.77)	406.23 (304.52,473.77)	13818378.97 (10901617.54,15512983.80)	299.15 (235.39,335.76)	−0.94 (−1.03, −0.85)
Deaths	415441.64 (318290.64,478418.06)	21.19 (16.39,24.27)	774163.86 (618791.55,878334.00)	16.51 (13.22,18.73)	−0.68 (−0.76, −0.60)
High body-mass index					
Measure	1990		2021		EAPC (95% CI)
	Number (95% UI)	ASR, per 100,000 (95% UI)	Number (95% UI)	ASR, per 100,000 (95% UI)	
Both					
DALYs	5666016.79 (4251059.02,7069737.58)	144.72 (106.21,182.76)	12551752.03 (9489123.61,15451016.13)	147.33 (109.06,183.45)	0.15 (0.10,0.21)
Deaths	240095.95 (168850.61,313373.98)	6.83 (4.37,9.32)	594899.05 (362924.21,804913.65)	7.21 (4.23,9.94)	0.33 (0.27,0.39)
Male					
DALYs	2414649.44 (1794252.02,3011391.35)	131.37 (96.12,166.66)	5646139.70 (4247052.19,6976634.91)	142.25 (104.32,179.41)	0.40 (0.33,0.47)
Deaths	93612.78 (66086.33,120397.78)	5.97 (3.74,8.25)	243060.39 (159714.60,323101.44)	6.69 (3.98,9.30)	0.55 (0.47,0.63)
Female					
DALYs	3251367.35 (2294580.51,4161209.41)	154.37 (107.15,198.32)	6905612.33 (5009097.33,8756180.55)	150.26 (109.60,190.15)	−0.04 (−0.09,0.01)
Deaths	146483.17 (97562.11,196209.61)	7.37 (4.60,10.12)	351838.67 (201115.85,494197.17)	7.53(4.36,10.53)	0.20 (0.15,0.25)

ASR, age-standardized rates; DALYs, disability-adjusted life years; UI, uncertainty interval; CI, confidence Interval; EAPC, estimated annual percentage change.

### Regional deaths and DALYs

**Figure 1:**
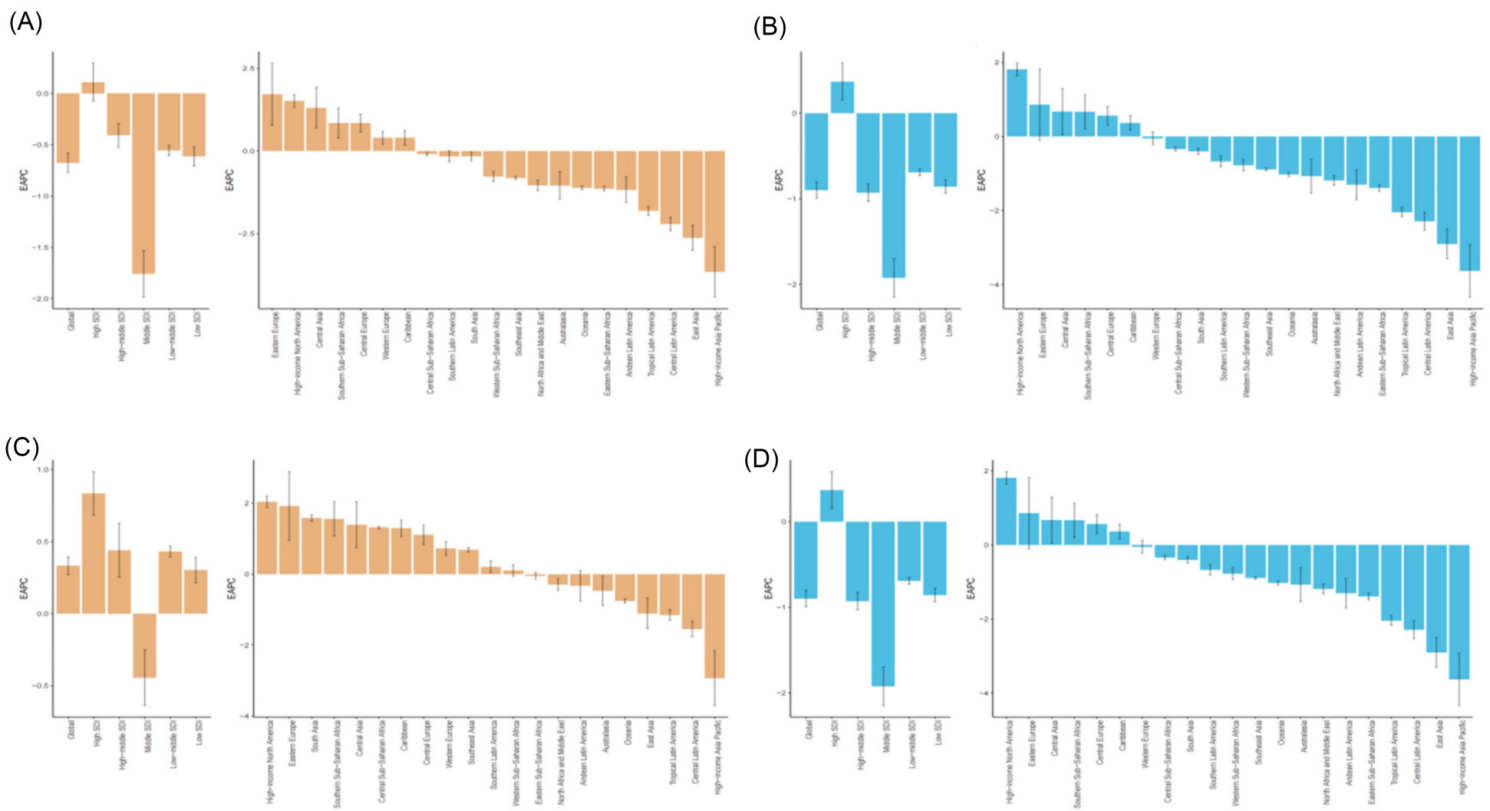
1990–2021 EAPC of the age-standardized rates for metabolic factors causing HHD. **(A)** EAPC of ASMR for HHD caused by HSBP in 21 regions and 5 SDI regions. **(B)** EAPC of ASDR for HHD caused by HSBP in 21 regions and 5 SDI regions. **(C)** EAPC of ASMR for HHD caused by high BMI in 21 regions and 5 SDI regions. **(D)** EAPC of ASDR for HHD caused by high BMI in 21 regions and 5 SDI regions.

At the regional level, in 2021, East Asia had the highest number of deaths [340,459.47 (95% UI: 235,390.26–437,522.23)] and DALYs [5,815,785.04 (95% UI: 4,183,000.54–7,412,101.31)] from HHD caused by HSBP, while Central Sub-Saharan Africa had the highest ASMR [66.27 (95% UI: 42.16–91.54)] and ASDR [1,212.96 (95% UI: 768.85–1,676.91)]. From 1990–2021, 7 regions saw an increase in ASMR, and 14 regions saw a decrease, while 6 regions had an increase in ASDR, and 15 regions saw a decrease. Among these, Eastern Europe had the largest increase in ASMR [EAPC: 1.71 (95% CI: 0.78–2.66)], and High-income North America had the largest increase in ASDR [EAPC: 2.31 (95% CI: 2.17–2.46)]. In contrast, High-income Asia Pacific showed the largest decrease in both ASMR [EAPC: −3.66 (95% CI: −4.41–−2.89)] and ASDR [EAPC: −3.63 (95% CI: −4.33–−2.92)] (Supplementary Table S1, Figure 1).

**Figure 2:**
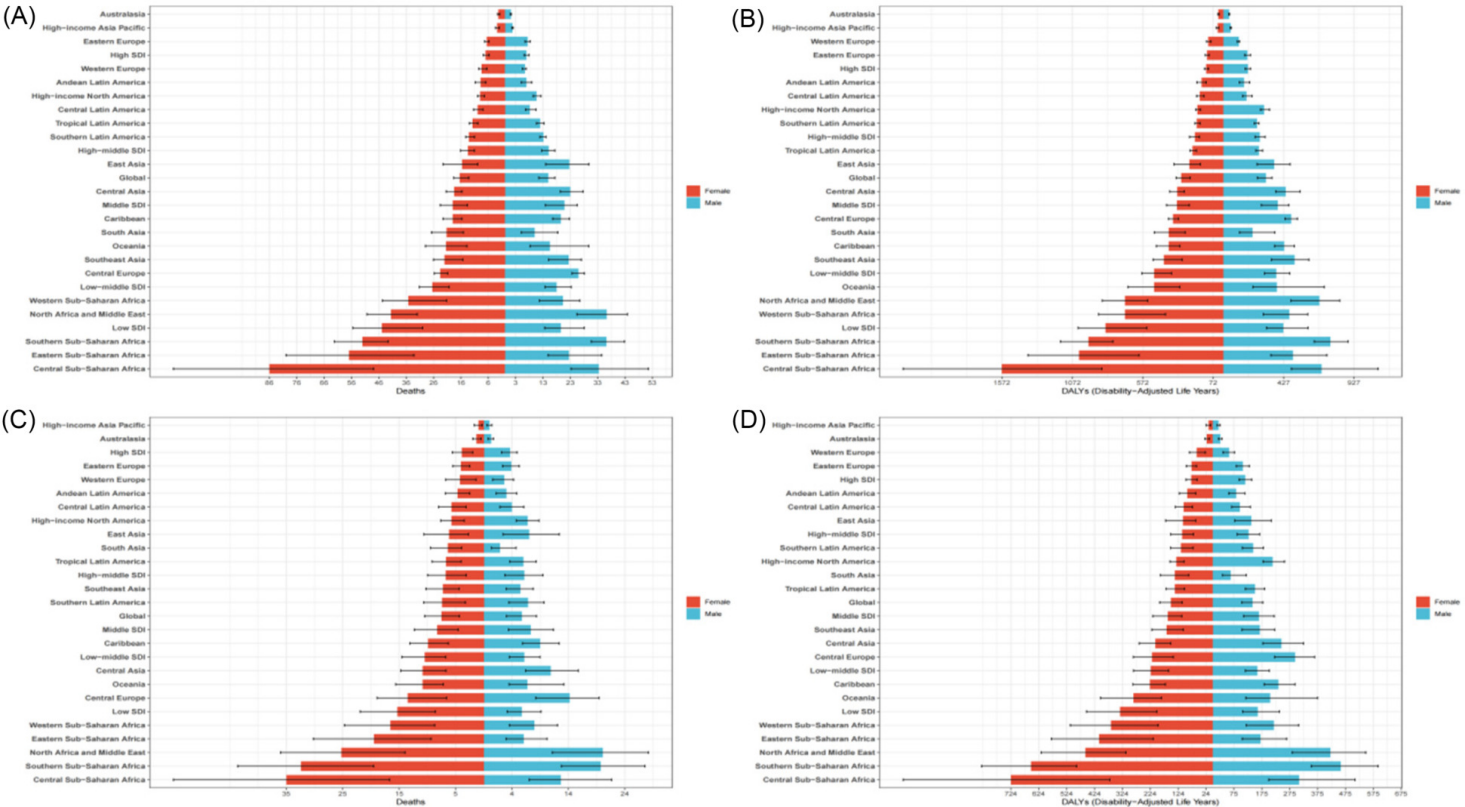
Gender differences in age-standardized rates of HHD caused by metabolic factors in 2021. **(A)** Gender differences in ASMR of HHD caused by HSBP in 21 regions. **(B)** Gender differences in ASDR of HHD caused by HSBP in 21 regions. **(C)** Gender differences in ASMR of HHD caused by high BMI in 21 regions. **(D)** Gender differences in ASDR of HHD caused by high BMI in 21 regions.

In 2021, East Asia also had the highest number of deaths [129,830.87 (95% UI: 67,188.11–204,255.03)] and DALYs [2,468,556.03 (95% UI: 1,553,649.60–3,558,544.21)] from HHD caused by high BMI, while Southern Sub-Saharan Africa had the highest ASMR [28.40 (95% UI: 17.52–37.54)] and ASDR [578.12 (95% UI: 437.99–722.53)]. From 1990–2021, 11 regions saw an increase in ASMR, and 10 regions saw a decrease, while 12 regions experienced an increase in ASDR, and 9 regions saw a decrease. Among these, High-income North America had the largest increase in both ASMR [EAPC: 2.04 (95% CI: 1.88–2.20)] and ASDR [EAPC: 2.31 (95% CI: 2.17–2.46)], whereas High-income Asia Pacific showed the largest decrease in both ASMR [EAPC: −2.93 (95% CI: −3.70–−2.16)] and ASDR [EAPC: −2.84 (95% CI: −3.54–−2.13)] (Supplementary Table S1, Figure 1). Notably, in 2021, the highest ASMR and ASDR due to HSBP and high BMI among females were observed in Central Sub-Saharan Africa, while for males, the highest ASMR and ASDR were observed in North Africa and the Middle East, and Southern Sub-Saharan Africa, respectively (Figure 2).

### National deaths and DALYs

**Figure 3:**
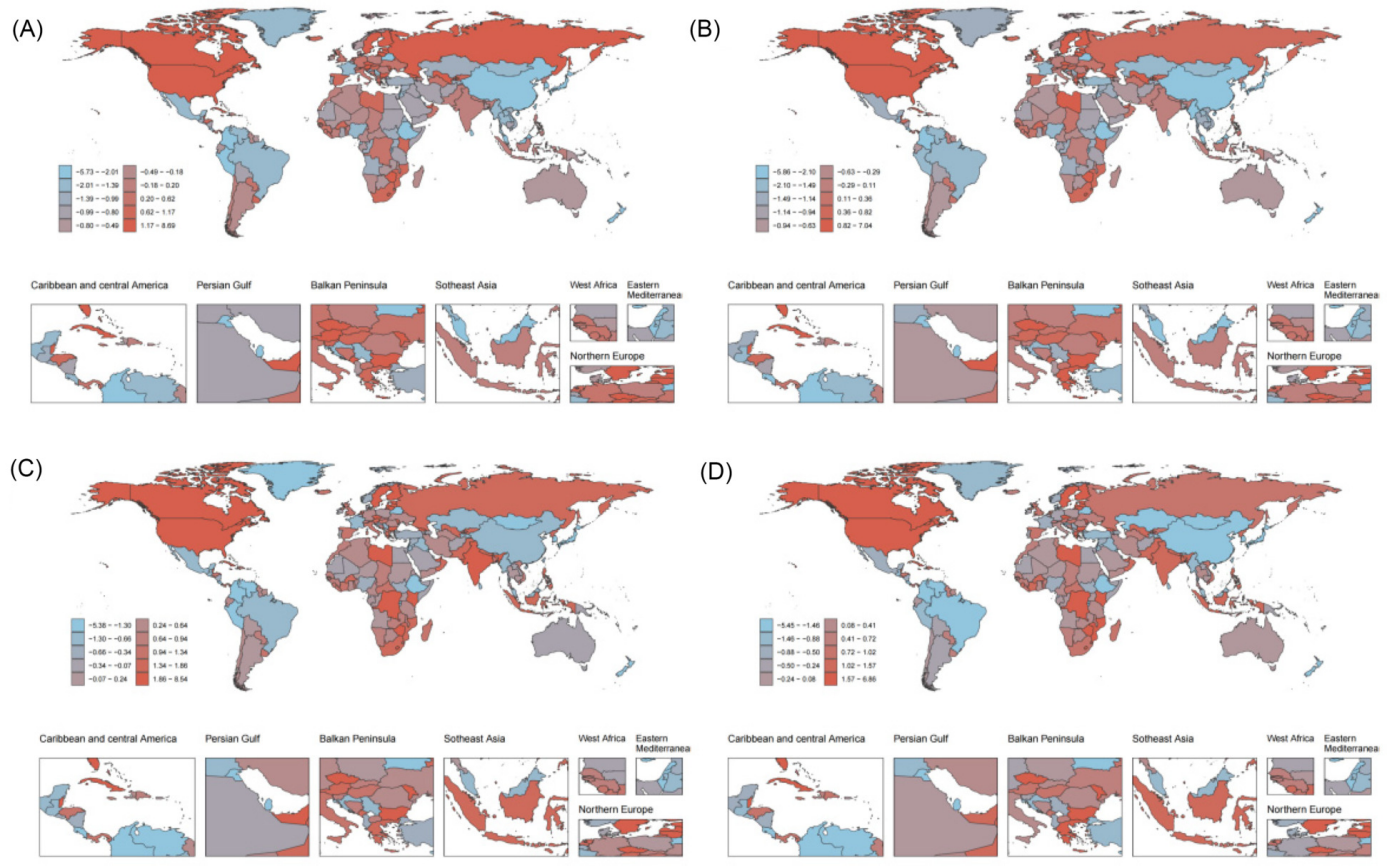
1990–2021 EAPC of the age-standardized rates for metabolic risk factors causing HHD are shown for 204 countries and regions. **(A)** EAPC of ASMR for HHD caused by HSBP in 204 countries and regions. **(B)** EAPC of ASDR for HHD caused by HSBP in 204 countries and regions. **(C)** EAPC of ASMR for HHD caused by high BMI in 204 countries and regions. **(D)** EAPC of ASDR for HHD caused by high BMI in 204 countries and regions.

At the national level, in 2021, China had the highest number of deaths [328,079.61 (95% UI: 224,196.25–424,637.69)] and DALYs [5,588,976.70 (95% UI: 3,974,289.38–7,176,387.41)] due to HHD caused by HSBP. On the other hand, Bulgaria had the highest age-standardized mortality rate (ASMR) [103.42 (95% UI: 89.56–117.24)] and age-standardized disability-adjusted life years rate (ASDR) [1,739.46 (95% UI: 1,489.32–1,992.26)]. From 1990–2021, the ASMR and ASDR for HHD caused by HSBP showed an increasing trend in 70 and 60 countries, respectively. In contrast, 134 countries experienced a decrease in ASMR, and 144 countries saw a decline in ASDR. Among them, Latvia showed the greatest increase in both ASMR [EAPC: 8.60 (95% CI: 7.31–9.92)] and ASDR [EAPC: 6.97 (95% CI: 5.77–8.18)], while Belarus had the greatest decrease in both ASMR [EAPC: −5.67 (95% CI: −6.78– −4.55)] and ASDR [EAPC: −5.80 (95% CI: −6.95– −4.63)] (Supplementary Table S2, Figures 3A,B).

In 2021, China also had the highest number of deaths [124,763.89 (95% UI: 63,333.52–198,270.70)] and DALYs [2,373,039.87 (95% UI: 1,485,461.40–3,462,005.03)] due to HHD caused by high BMI. The country with the highest ASMR was Botswana [59.71 (95% UI: 34.53–83.60)], while Bulgaria had the highest ASDR [1,083.92 (95% UI: 779.93–1,396.41)]. From 1990–2021, the ASMR and ASDR for HHD caused by high BMI showed an increasing trend in 115 and 100 countries, respectively. In contrast, 89 countries experienced a decrease in ASMR, and 104 countries saw a decrease in ASDR. Among them, Latvia again showed the greatest increase in both ASMR [EAPC: 8.46 (95% CI: 7.18–9.76)] and ASDR [EAPC: 6.79 (95% CI: 5.60–7.99)], while Belarus had the greatest decrease in both ASMR [EAPC: −5.33 (95% CI: −6.50– −4.15)] and ASDR [EAPC: −5.40 (95% CI: −6.59– −4.19)] (Supplementary Table S2, Figures 3C,D).

### Burden of HHD caused by metabolic factors based on age and sex

In 2021, the age groups with the highest number of deaths and DALYs for HSBP-related HHD were 85–89 years for women and 70–74 years for men. Additionally, both women and men showed an increasing trend in ASMR and ASDR with advancing age. Notably, from age 64 onward, women had higher numbers of deaths, DALYs, ASMR, and ASDR than men, indicating that women may face higher cardiovascular risks during aging.

**Figure 4:**
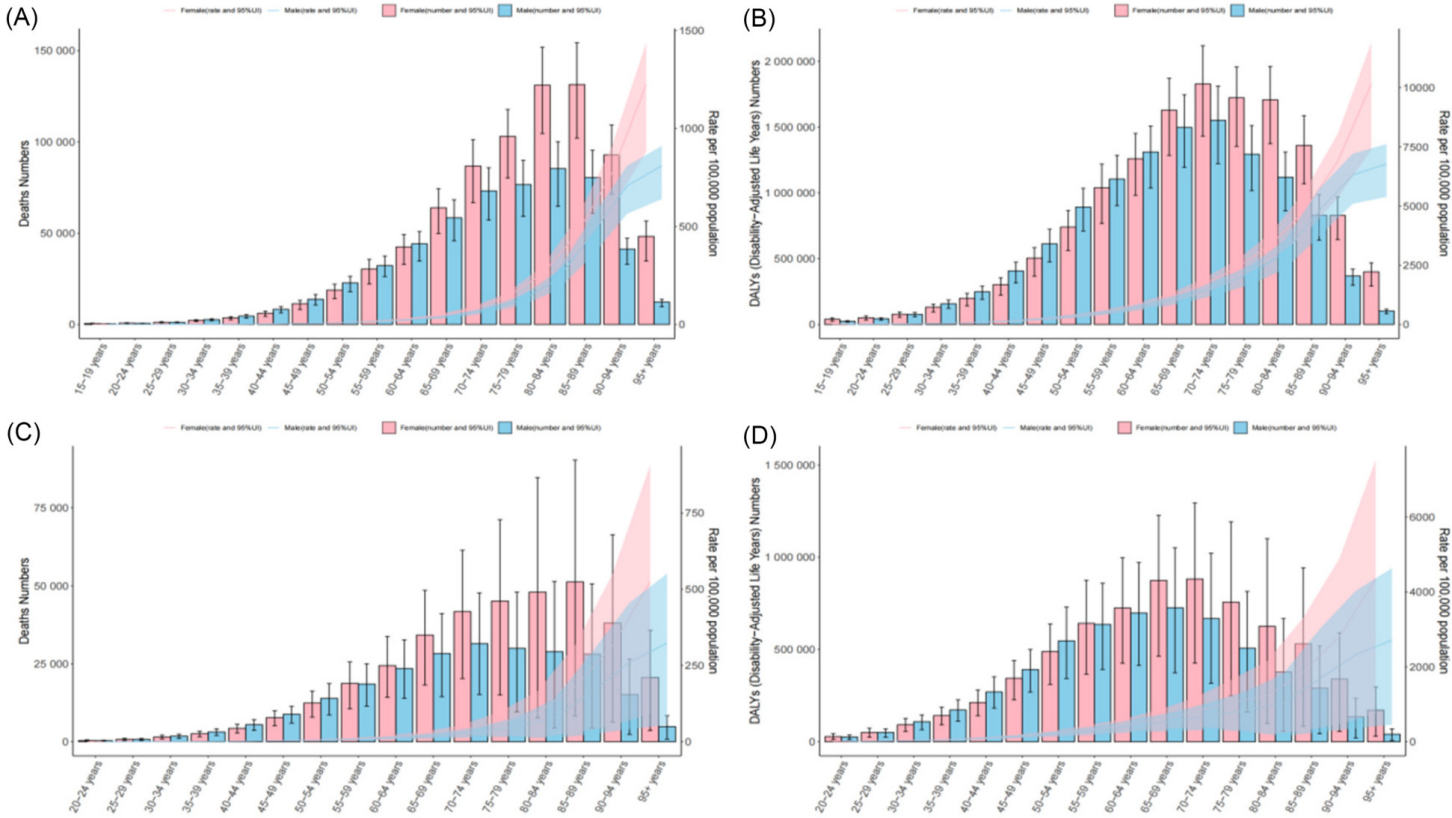
The age-specific numbers and rates of deaths and DALYs from HHD caused by HSBP and high BMI in 2021, stratified by sex. **(A)** The age-specific numbers and rates of deaths from HHD caused by HSBP. **(B)** The age-specific numbers and rates of DALYs from HHD caused by HSBP. **(C)** The age-specific numbers and rates of deaths from HHD caused by high BMI. **(D)** The age-specific numbers and rates of DALYs from HHD caused by high BMI.

For High BMI-related HHD, the age groups with the highest number of deaths and DALYs for women were again 85–89 years and 70–74 years, while for men, the highest burden was observed in the 70–74 years and 65–69 years age groups. Both women and men showed increasing ASMR and ASDR as age increased due to high BMI. Particularly, after age 54, women consistently had higher numbers of deaths, DALYs, ASMR, and ASDR than men, further highlighting the unique cardiovascular risks women face with higher BMI-related diseases (Figure 4).

### Burden of HHD caused by metabolic factors based on SDI

In 2021, Middle SDI regions had the highest number of deaths [461,701.61 (95% UI: 348,619.59, 545,738.73)] and DALYs [8,930,223.42 (95% UI: 6,899,892.63, 10,419,863.71)] for HSBP-related HHD. Conversely, Low SDI regions had the highest ASMR [33.56 (95% UI: 24.63, 40.51)] and ASDR [640.45 (95% UI: 449.91, 784.43)] for HSBP-related HHD. From 1990–2021, the ASMR for High SDI regions showed an upward trend (EAPC: 0.11, 95% CI: −0.07, 0.29), while ASMR and ASDR in other SDI regions generally showed a downward trend, with Middle SDI regions showing the largest decline in both ASMR (EAPC: −1.76, 95% CI: −1.98, −1.53) and ASDR (EAPC: −1.92, 95% CI: −2.15, −1.70) (Supplementary Table S1, Figure 1).

For High BMI-related HHD, in 2021, Middle SDI regions again had the highest number of deaths [196,804.87 (95% UI: 122,493.59, 276,060.94)] and DALYs [4,255,946.72 (95% UI: 3,088,318.19, 5,431,403.13)]. Low SDI regions also had the highest ASMR [11.27 (95% UI: 6.94, 15.88)] and ASDR [249.65 (95% UI: 164.84, 328.72)] for High BMI-related HHD. From 1990–2021, Middle SDI regions showed a downward trend in ASMR (EAPC: −0.44, 95% CI: −0.64, −0.25), while other SDI regions had an increasing trend in ASMR. Among these, High SDI regions showed the most significant increase in ASMR (EAPC: 0.44, 95% CI: −0.64, −0.25). Additionally, High-middle SDI and Middle SDI regions saw a decline in ASDR, while other regions experienced an increase. The regions with the largest decline in ASDR were Middle SDI, and the highest increase was in High SDI (Supplementary Table S1, Figure 1).

**Figure 5:**
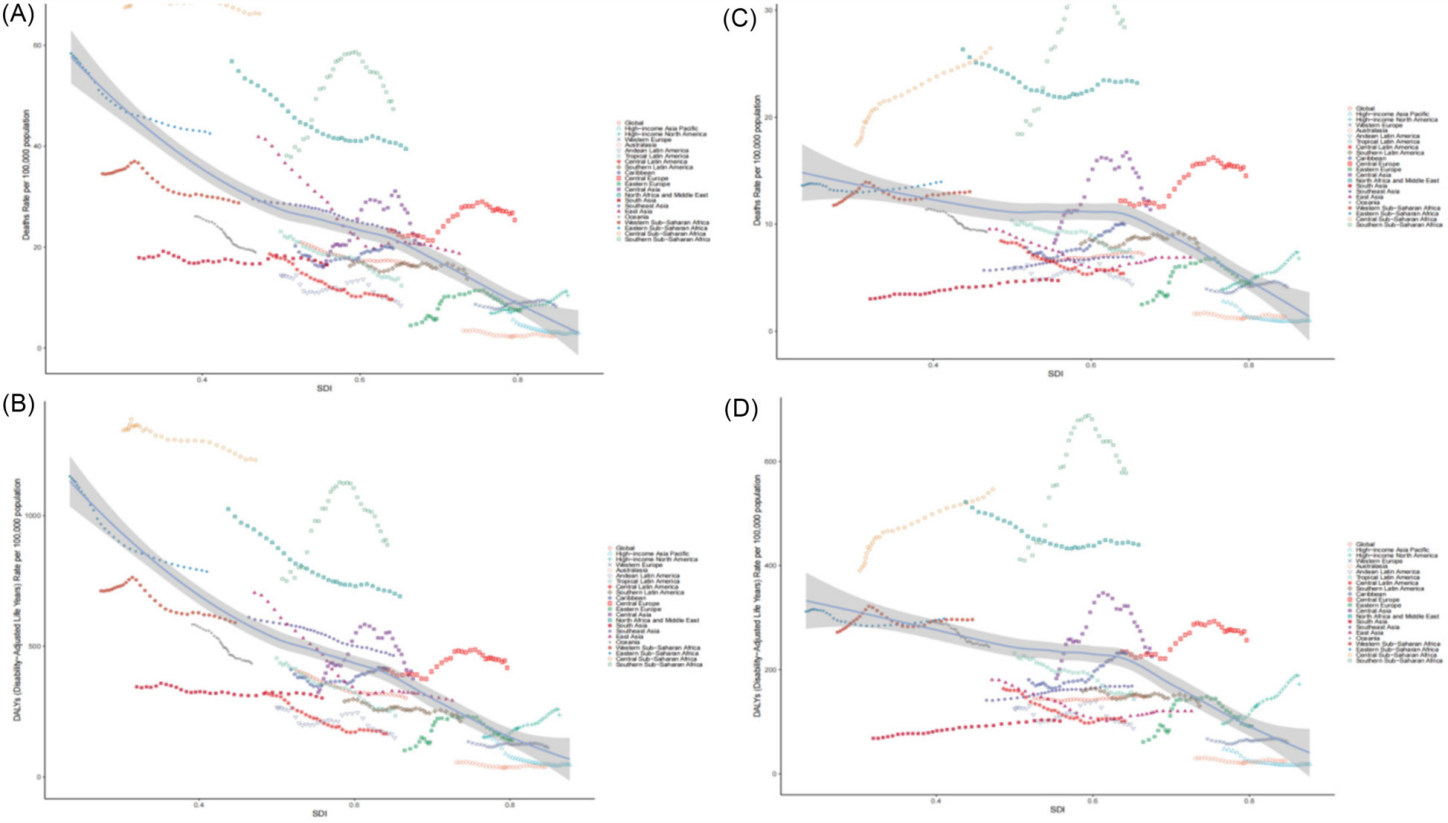
The age-standardized rates of HHD caused by HSBP and high BMI across 21 global regions from 1990 to 2021, stratified by the SDI. **(A)** ASMR for HHD caused by HSBP. **(B)** ASDR for HHD caused by HSBP. **(C)** ASMR for HHD caused by high BMI. **(D)** ASDR for HHD caused by high BMI.

**Figure 6:**
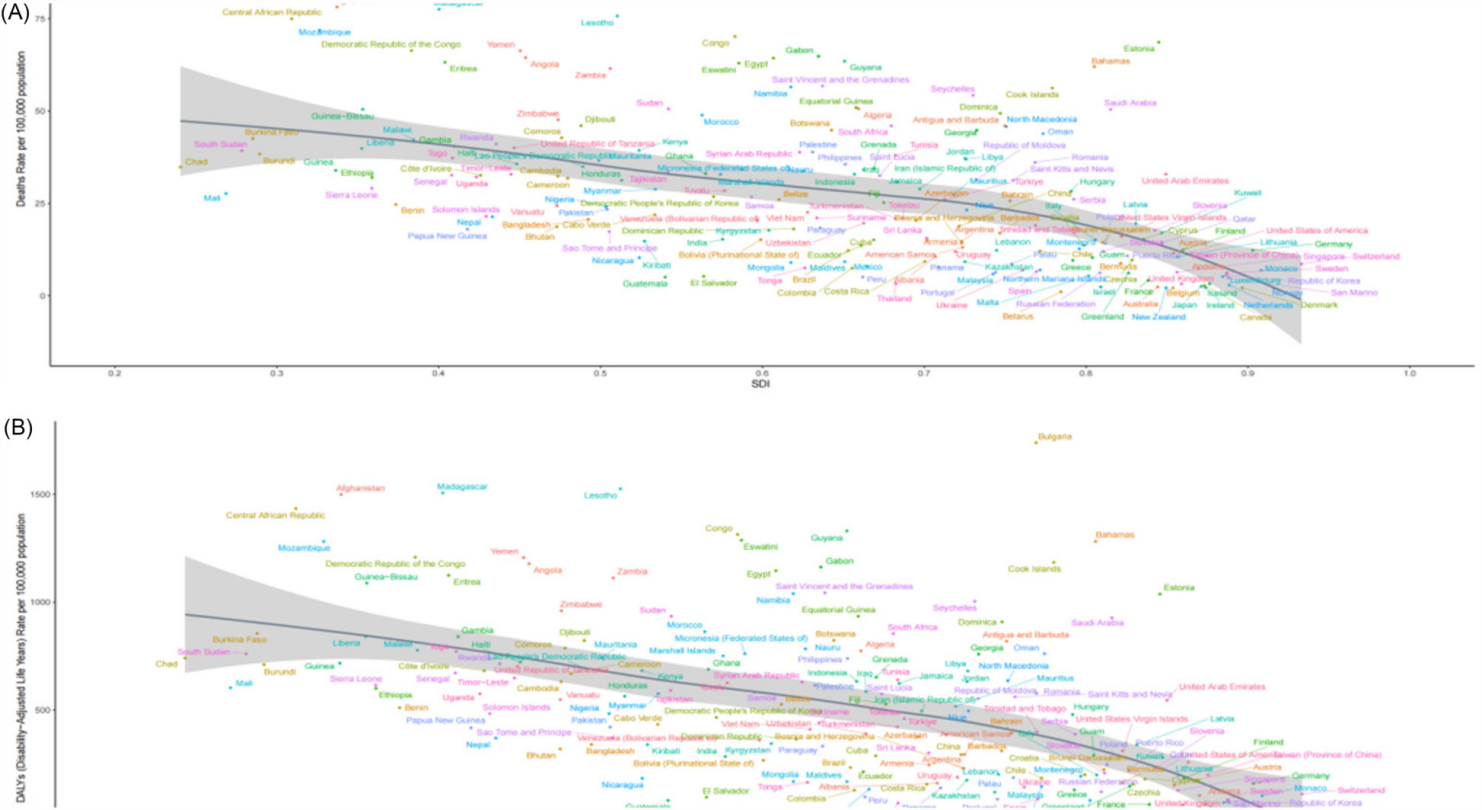
The age-standardized rates of HHD caused by HSBP and across 204 countries and territories from 1990 to 2021, stratified by the SDI. **(A)** ASMR for HHD caused by HSBP. **(B)** ASDR for HHD caused by HSBP.

From 1990–2021, in Low SDI regions, the ASMR and ASDR caused by HSBP showed a significant negative correlation with SDI, while the ASMR and ASDR caused by High BMI showed a generally positive correlation with SDI. In Middle SDI regions, with some exceptions (such as Southern Sub-Saharan Africa, Central Asia, Central Europe, Eastern Europe, and Caribbean), the ASMR and ASDR caused by HSBP showed a negative correlation with SDI, while those caused by High BMI showed a positive correlation in most regions (such as Andean Latin America, Central Latin America, Tropical Latin America, North Africa and the Middle East, East Asia, and Oceania). In High SDI regions, except for High-income North America, the ASMR and ASDR caused by both HSBP and High BMI had a significant negative correlation with SDI (Figure 5). At the national level in 2021, there was a significant negative correlation between ASMR and ASDR caused by HSBP and SDI, while in Low and Middle SDI regions, the ASMR and ASDR caused by High BMI generally showed a positive correlation with SDI. In High SDI regions, however, the correlation was negative (Figure 6, Supplementary Figure S1).

## Discussion

This study systematically evaluated the evolving burden of HHD attributable to HSBP and BMI from 1990–2021, with a focus on the central role of metabolic risk factors in HHD progression and their heterogeneous distribution across global regions. The findings show that the proportion of HHD deaths attributable to metabolic factors increased from 13.38% in 1990 to 16.88% in 2021, indicating that the metabolic burden is continuously driving up the disease burden related to HHD (Supplementary Figure S2). Among various metabolic risk factors, HSBP and high BMI were identified as the primary contributors to HHD (11). HSBP increases cardiac afterload, inducing left ventricular hypertrophy, myocardial remodeling, and arteriosclerosis, which can further lead to myocardial ischemia and heart failure (11). High BMI, on the other hand, promotes chronic low-grade inflammation, insulin resistance, and dysregulated lipid metabolism, accelerating metabolic injury and functional decline of the myocardium (13–15). More importantly, the interaction between hypertension and obesity may form a pathological positive feedback loop, mutually exacerbating cardiovascular stress and significantly increasing the risk of HHD (22, 23). Previous studies have shown that the burden of cardiovascular disease due to metabolic factors exhibits significant temporal and spatial heterogeneity, closely linked to urbanization, westernization of dietary patterns, reduced physical activity, and disparities in healthcare coverage and access (24–26). Therefore, targeted interventions addressing these two major metabolic risk factors—especially strengthening early screening, lifestyle modification, and pharmacological control in high-burden populations—will be critical strategies for alleviating the global burden of HHD and reducing cardiovascular mortality in the future.

The study found that from 1990–2021, deaths from HHD attributable to HSBP and BMI increased by 86.62% and 147.78%, respectively, while DALYs rose by 64.58% and 121.53%. Despite these increases, the ASMR and ASDR associated with HSBP showed a downward trend globally, reflecting significant achievements in hypertension control in certain countries. For example, the Million Hearts initiative in the United States, led by the CDC and CMS, implemented evidence-based strategies—including optimized hypertension screening, enhanced primary care services, and integration of clinical decision support into electronic health records. The initiative has significantly improved blood pressure control rates and contributed to the reduction of HHD and associated cardiovascular events (27). Similarly, Finland implemented the comprehensive North Karelia Project, which included nutritional interventions, smoking and salt reduction campaigns, and community-based health education, successfully lowering population blood pressure levels and cardiovascular mortality (28, 29). In contrast, ASMR and ASDR related to high BMI have continued to rise, highlighting the escalating global obesity epidemic, particularly in low- and middle-income countries. The rapid spread of obesity is offsetting some of the gains made in hypertension prevention and is amplifying the overall cardiovascular disease burden. Among individuals with both hypertension and obesity, this risk is further compounded. Alarmingly, if current trends persist, obesity may surpass hypertension as the leading driver of HHD in the future. Effective weight management, increased physical activity, and dietary optimization are essential strategies to reduce the burden of obesity-related HHD (30, 31). For example, several U.S. states have enacted legislation to limit sugary beverage consumption, implemented school-based nutrition programs, and strengthened physical education, which have collectively helped curb the rise in adolescent obesity rates (32, 33). Therefore, future public health efforts should prioritize early identification and intervention for obesity, establish more systematic weight control frameworks at the policy level, and promote multi-level, comprehensive global interventions. These measures are especially crucial in resource-limited developing regions where obesity is rising rapidly, in order to effectively mitigate the health and socioeconomic burden posed by HHD.

In 2021, the burden of HHD attributable to HSBP and high BMI varied significantly across global regions and countries. For instance, East Asia recorded the highest number of deaths and DALYs related to HSBP-induced HHD worldwide, reflecting the considerable challenges in hypertension management within the region. Despite progress in public health policy, rapid urbanization and lifestyle changes have led to a continued rise in hypertension, severely impacting cardiovascular health (34). In contrast, Central Sub-Saharan Africa reported the highest ASMR and ASDR, highlighting severe issues linked to inadequate healthcare resources and insufficient hypertension control. Between 1990 and 2021, ASMR increased in seven regions and ASDR in six, with Eastern Europe and High-income North America experiencing the most pronounced rises. These trends may be associated with poor dietary habits, increased psychosocial stress, and a rise in sedentary behavior. In contrast, the High-income Asia Pacific region saw significant declines in both ASMR and ASDR, reflecting the positive impact of early interventions and adequate healthcare resources. Regarding HHD burden related to high BMI, East Asia again ranked first in terms of deaths and DALYs, primarily due to the rapid spread of obesity. This trend is particularly alarming in low- and middle-income countries, where obesity has emerged as a major public health challenge (35). Southern Sub-Saharan Africa reported the highest ASMR and ASDR for BMI-related HHD, underscoring the urgency of obesity prevention in this region. Although High-income North America has abundant healthcare resources, its high obesity prevalence has led to marked increases in ASMR and ASDR, further exacerbating the burden of HHD.

In 2021, China recorded the highest number of deaths and DALYs from HHD attributable to HSBP and high BMI, highlighting a severe public health challenge under the dual pressure of accelerating urbanization and population aging. Although recent progress has been made in blood pressure and weight control through health education, the establishment of tiered healthcare systems, and improved access to antihypertensive medications, the nationwide prevalence of hypertension and obesity remains high, continuing to burden the national healthcare system (36). Currently, China faces three core challenges in HHD prevention and control (36): First, the uneven distribution of healthcare resources between urban and rural areas means that primary care systems are not yet equipped to conduct widespread early screening and long-term management of chronic diseases. Second, unhealthy lifestyles, including high-salt and high-fat diets combined with sedentary behavior, are accelerating the development of metabolic disorders. Third, the impact of individual genetic differences on treatment response has not been fully integrated into current management strategies. Previous studies have suggested that certain genetic polymorphisms related to blood pressure regulation—such as PHACTR1 (37) and PTGER3 (38)—may affect the efficacy of antihypertensive drugs, underscoring the urgent need for personalized interventions based on genetic background. By contrast, Bulgaria had the highest ASMR and ASDR for HHD attributable to HSBP, as well as the highest ASDR for BMI-related HHD globally in 2021. This reflects structural weaknesses in its healthcare system during economic transition, including high unemployment, low income levels, and inadequate access to medical resources (39). Between 1990 and 2021, seven of the ten countries with the greatest increases in ASMR and ASDR were located in Eastern Europe. The rising burden of HHD in the region has been driven by population aging, excessive sodium intake, poor dietary patterns, physical inactivity, and insufficient chronic disease management (40–42). Furthermore, epigenetic studies have shown that populations under long-term socioeconomic stress are more likely to develop abnormal DNA methylation (43, 44) and histone modifications (45, 46), which can induce chronic inflammation and exacerbate cardiometabolic disorders. Although some Eastern European countries have implemented public health measures such as family doctor systems and salt reduction campaigns, inconsistent policy implementation and widening urban-rural disparities remain key barriers. Moving forward, the region should prioritize early screening and comprehensive intervention for hypertension and obesity, enhance health behavior modification and chronic disease literacy, and integrate genetic and epigenetic profiling into risk stratification and precision management. These efforts will be vital to curbing the rising burden of HHD and advancing global cardiovascular health governance.

In 2021, middle-SDI regions reported the highest number of deaths and DALYs due to HHD attributable to HSBP and high BMI, indicating substantial challenges in cardiovascular disease prevention and control in these areas. Meanwhile, low-SDI regions exhibited the highest ASMR and ASDR, reflecting more severe issues in disease management due to limited medical resources and inadequate healthcare services. Between 1990 and 2021, ASMR and ASDR showed an upward trend in high-SDI regions, whereas most other SDI regions experienced declines. The most notable decreases in ASMR and ASDR occurred in middle-SDI regions, likely due to recent improvements in hypertension and obesity management. Interestingly, in low-SDI regions, ASMR and ASDR associated with HHD due to HSBP were significantly negatively correlated with SDI, whereas the burden related to BMI showed an overall positive correlation. This suggests that insufficient hypertension management in low-SDI areas has greatly aggravated the disease burden, while BMI-related burden tends to increase as SDI rises. In middle-SDI regions, the HHD burden from HSBP was generally negatively correlated with SDI, whereas the burden from high BMI was positively correlated, reflecting regional disparities in cardiovascular risk factor management and levels of economic development. In high-SDI regions, except for high-income North America, both HSBP- and BMI-related ASMR and ASDR were significantly negatively correlated with SDI. At the national level, data showed a negative correlation between SDI and the HHD burden attributable to HSBP, while BMI-related burden was positively correlated with SDI in low- and middle-SDI countries and negatively correlated in high-SDI countries. This indicates that high-income regions are relatively more effective in obesity control, whereas low- and middle-SDI countries continue to face serious challenges in addressing the obesity epidemic.

In 2021, the burden of HHD attributable to HSBP and high BMI exhibited significant heterogeneity across sex and age groups. Overall, with increasing age, both males and females showed a consistent upward trend in the number of deaths, DALYs, ASMR, and ASDR. However, it is noteworthy that although ASMR and ASDR were similar between sexes, women consistently exhibited higher absolute numbers of deaths and DALYs than men, suggesting that older women may face more complex and severe health challenges related to HHD outcomes. This gender difference likely results from a combination of biological, metabolic, and social factors. On one hand, postmenopausal women experience a marked decline in estrogen levels, leading to the loss of cardiovascular protective effects (47). On the other hand, older women are more prone to visceral fat accumulation, insulin resistance, and metabolic syndrome (48), all of which intensify metabolic stress on the cardiovascular system. Furthermore, women may face structural barriers in chronic disease management, such as limited access to healthcare, poor adherence to medical treatment, lower health awareness, and insufficient coverage of lifestyle interventions (49, 50), all of which may contribute to a sustained disadvantage in disease burden. By contrast, in some regions, the ASMR for men has increased at a faster rate, likely linked to a higher prevalence of unhealthy behaviors. Men are generally more exposed than women to high-salt and high-fat diets, tobacco and alcohol use, and lack of regular physical activity—factors that play key roles in the onset and progression of cardiovascular diseases (51, 52). In addition, in certain cultural contexts, men tend to be more passive in seeking medical care, and delayed healthcare utilization may worsen disease progression and outcomes. Therefore, the development of sex-specific intervention strategies is essential. For women, particularly those in the menopausal and postmenopausal stages, early identification and comprehensive management of metabolic risk factors should be strengthened. Integrated strategies that include screening for hypertension and obesity, personalized health education, and lifestyle interventions should be promoted. For men, increasing health awareness, improving lifestyle habits, and encouraging proactive healthcare-seeking behaviors are crucial. Interventions should focus on modifiable risk factors such as smoking, alcohol consumption, diet, and physical inactivity to reduce HHD-related mortality and disability among men.

### Policy recommendations

This study systematically analyzed the global burden trends of HHD attributable to HSBP and high BMI from 1990–2021, and based on the findings, the following policy recommendations are proposed: First, strengthen hypertension prevention and control systems, with a particular focus on low- and middle-income countries and high-burden regions such as sub-Saharan Africa. Efforts should center on improving early screening rates and the standardization of diagnosis and treatment through primary healthcare systems, ensuring stable and affordable access to essential antihypertensive medications. At the same time, community-based health education should be enhanced to raise public awareness of risk factors and improve self-management capabilities to prevent the early accumulation of hypertension-related risks. Second, address the global obesity epidemic in a systematic manner, particularly in high-income and rapidly urbanizing areas. A structured, multi-level intervention strategy is needed, including restrictions on the availability and consumption of high-energy foods, strengthening of nutritional labeling and food policy regulations, promotion of adolescent nutrition and physical activity, and the implementation of tiered weight management services, all aimed at reducing cardiovascular risk linked to elevated BMI. Third, promote gender- and age-sensitive interventions to reduce health inequalities. It is recommended to strengthen early screening for metabolic syndrome and implement combined interventions targeting blood pressure and weight in postmenopausal women, while intensifying integrated interventions in young and middle-aged men to address high-risk behaviors such as high salt intake, tobacco and alcohol use, and sedentary lifestyles, thereby promoting the adoption of healthy habits. Fourth, optimize the allocation of global health resources to enhance chronic disease prevention and control capacity in low-SDI regions. This should be achieved through multilateral cooperation mechanisms that provide financial aid and technical support to address critical gaps in screening, diagnosis, and management of hypertension and obesity, ensuring more equitable and efficient distribution of global public health resources. Fifth, recognize the preventive and therapeutic potential of traditional medicine and promote its standardized integration into chronic disease management. Drawing on practical experience with traditional Chinese medicine (TCM) in HHD intervention, herbal medicines such as Salvia miltiorrhiza (Danshen) (53), Pueraria lobata (Gegen) (54), Scutellaria baicalensis (Huangqin) (55), and Prunella vulgaris (Xiakucao) (56) have shown certain benefits in regulating blood pressure and body weight. These effects may be mediated through anti-inflammatory, antioxidant, endothelial function-regulating, and lipid metabolism-modulating mechanisms, achieving multi-target synergistic intervention. Although current evidence mainly comes from small-sample studies, in resource-limited settings with poor patient adherence, TCM holds promise as a valuable supplement for high-risk population management. Moving forward, large-scale, multi-center, high-quality clinical studies should be encouraged to facilitate the scientific integration and standardized application of TCM within comprehensive HHD intervention systems.

### Limitations and future research directions

This study, based on the GBD 2021 database, comprehensively assessed the gender, age, and SDI-related changes in the burden of HHD but has several limitations. Firstly, the GBD model relies on the integration of multiple data sources, which may be subject to bias and uncertainty, especially in low- and middle-income countries where data quality control may be inadequate. Secondly, this study did not include the synergistic effects of other metabolic risk factors (such as diabetes and hyperlipidemia) on the burden of HHD, which may lead to an underestimation of the complex interactions of multiple factors. Therefore, future research should explore the combined effects of multiple metabolic risk factors on the burden of HHD, particularly in high-risk regions and populations, to better understand the interactions between risk factors. This will provide crucial support for the development of more effective public health policies.

## Conclusions

This study revealed the changing global burden of HHD caused by HSBP and high BMI, highlighting the persistent impact of metabolic risk factors on the burden of HHD. To address the global challenge of HHD, it is essential to strengthen the prevention and management of hypertension and obesity, particularly in low- and middle-income countries. Additionally, attention must be given to gender and regional differences, and targeted health intervention strategies should be developed for different groups to reduce the disease risks caused by metabolic factors.

## Data Availability

The datasets presented in this study can be found in online repositories. The names of the repository/repositories and accession number(s) can be found below: https://vizhub.healthdata.org/gbd-results/.
