# Supplementary material for: Burden of hypertensive heart disease attributed to metabolic factors from 1990 to 2021 at global, regional, and national levels: an analysis of the global burden of disease study 2021
Source: Front Cardiovasc Med. 2025 May 29;12:1572392. doi: 10.3389/fcvm.2025.1572392 (PMC12158952; doi:10.3389/fcvm.2025.1572392)
Supplement: Supplementary file 1 [file Supplementaryfile1.pdf]

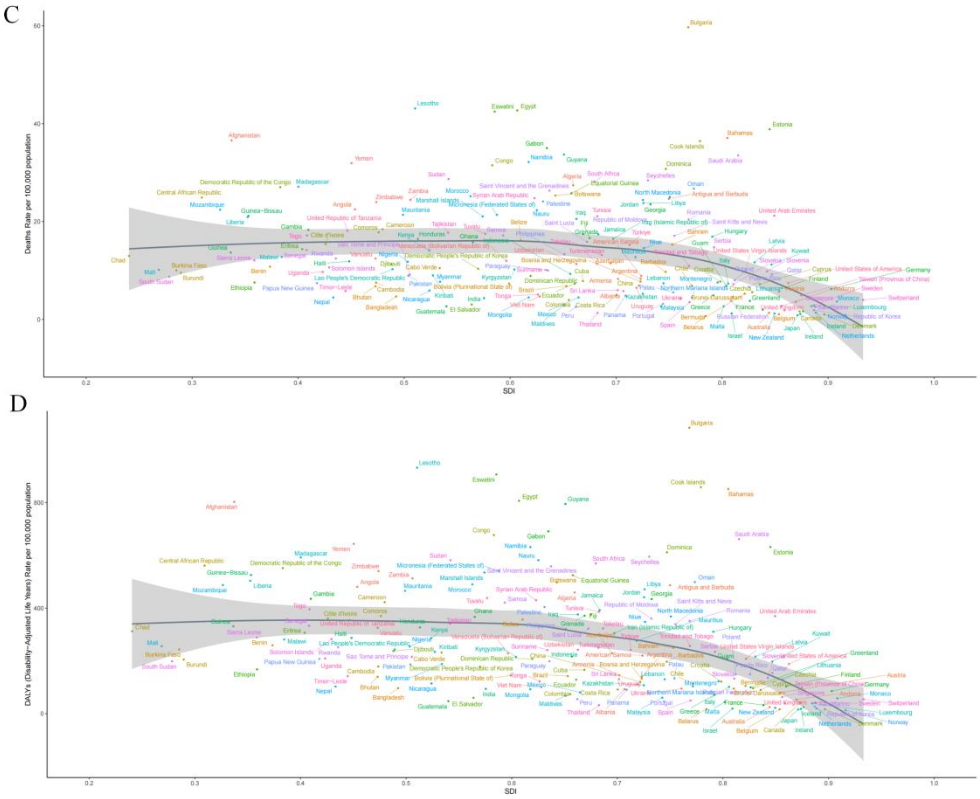

Supplementary Figure 1 The age-standardized rates of HHD caused by high BMI across 204 countries and territories from 1990 to 2021, stratified by the SDI. A. ASMR for HHD caused by high BMI. B. ASDR for HHD caused by high BMI.

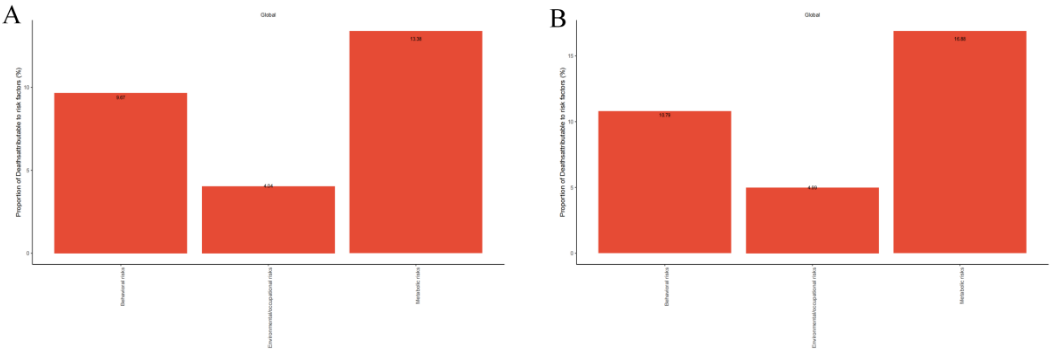

Supplementary Figure 2 Attributable risk proportion of HHD caused by metabolic factors globally in 1990 and 2021. A. Attributable risk proportion of HHD caused by metabolic factors globally in 1990. B. Attributable risk proportion of HHD caused by metabolic factors globally in 2021.
